# Comparing Methods for Prioritising Protected Areas for Investment: A Case Study Using Madagascar’s Dry Forest Reptiles

**DOI:** 10.1371/journal.pone.0132803

**Published:** 2015-07-10

**Authors:** Charlie J. Gardner, Christopher J. Raxworthy, Kristian Metcalfe, Achille P. Raselimanana, Robert J. Smith, Zoe G. Davies

**Affiliations:** 1 Durrell Institute of Conservation and Ecology (DICE), School of Anthropology and Conservation, University of Kent, Canterbury, Kent, United Kingdom; 2 WWF Madagascar and Western Indian Ocean Programme Office, BP 738, Antananarivo 101, Madagascar; 3 Department of Herpetology, American Museum of Natural History, New York, New York, United States of America; 4 Centre for Ecology and Conservation, University of Exeter, Penryn, Cornwall, United Kingdom; 5 Environment and Sustainability Institute, University of Exeter, Penryn Campus, Cornwall, United Kingdom; 6 Association Vahatra, BP 3972, Antananarivo 101, Madagascar; 7 Département de Biologie Animale, Faculté des Sciences, BP 906, Université d’Antananarivo, Antananarivo 101, Madagascar; Point Blue Conservation Science, UNITED STATES

## Abstract

There are insufficient resources available to manage the world’s existing protected area portfolio effectively, so the most important sites should be prioritised in investment decision-making. Sophisticated conservation planning and assessment tools developed to identify locations for new protected areas can provide an evidence base for such prioritisations, yet decision-makers in many countries lack the institutional support and necessary capacity to use the associated software. As such, simple heuristic approaches such as species richness or number of threatened species are generally adopted to inform prioritisation decisions. However, their performance has never been tested. Using the reptile fauna of Madagascar’s dry forests as a case study, we evaluate the performance of four site prioritisation protocols used to rank the conservation value of 22 established and candidate protected areas. We compare the results to a benchmark produced by the widely-used systematic conservation planning software Zonation. The four indices scored sites on the basis of: i) species richness; ii) an index based on species’ Red List status; iii) irreplaceability (a key metric in systematic conservation planning); and, iv) a novel Conservation Value Index (*CVI*), which incorporates species-level information on endemism, representation in the protected area system, tolerance of habitat degradation and hunting/collection pressure. Rankings produced by the four protocols were positively correlated to the results of Zonation, particularly amongst high-scoring sites, but *CVI* and *Irreplaceability* performed better than *Species Richness* and the *Red List Index*. Given the technological capacity constraints experienced by decision-makers in the developing world, our findings suggest that heuristic metrics can represent a useful alternative to more sophisticated analyses, especially when they integrate species-specific information related to extinction risk. However, this can require access to, and understanding of, more complex species data.

## Introduction

Conservation is severely under-resourced globally [1, 2], but particularly in tropical developing countries where biodiversity is concentrated [3, 4], so interventions must be prioritised to ensure maximum impact. The principal strategy for conserving biodiversity is the establishment of protected areas, which now cover almost 13% of the world’s land surface [5, 6]. However, these areas vary greatly in the value of their constituent biodiversity [7, 8], having largely been established in landscapes where opportunity costs have been lowest [9–11], rather than the most important for conservation [12, 13].

Although protected areas can successfully reduce the pressures that threaten biodiversity [10, 14–16], their performance is largely dependent on investment in active management. Thus, even modest increases in funding could substantially improve protected area effectiveness [17]. Ongoing deforestation and forest degradation in protected areas worldwide (e.g. [18–20]) suggests that there are more sites than can be financed and managed optimally in many countries, and that protected areas are under-resourced at the global level [21]. Given the huge variation in the contribution of protected areas to global biodiversity conservation [7, 8], and the fact that conservation goals are achieved most efficiently if available investment is targeted preferentially towards the most important sites [22], it follows that the optimal allocation of conservation resources will necessitate triage [23]. This does not, however, imply that low-value or underperforming protected areas should be degazetted, since even paper parks (which exist in legislation but lack any management [24]) may be somewhat effective at conserving biodiversity [17].

Decisions regarding the selection of priority areas for investment from within existing protected area networks should be evidence-based. However, such choices are almost always made by state-mandated protected area management agencies, non-governmental organisations and/or conservation funding bodies (henceforth decision-makers), actors who do not tend to make sufficient, appropriate or systematic use of scientific tools and approaches in decision-making [25–29]. Indeed, their priorities may be influenced by the (often implicit) values held by individuals and organisations [30–33], rather than rational, explicit methods, and this may lead to suboptimal results.

A range of metrics and approaches can be used to provide an evidence base for protected area prioritisation and triage, including the sophisticated software tools developed for systematic conservation planning and assessments (i.e. to inform the design of protected area portfolios where the representation and persistence of biodiversity are maximised for least cost) [34]. However, the uptake and use of such tools by decision-makers is limited by the need for specific training (which can be expensive and time-consuming), data availability, institutional support and institutional memory, since those receiving training are often moved into higher-level roles as a result [35, 36]. Even in contexts where significant investments have been made in building capacity for the use of such technological tools (such as over the last decade in Madagascar), barriers to their uptake by decision-makers remain; as a result, prioritisation decisions continue to be carried out based on simple measures such as richness of threatened species (e.g. [37, 38]).

Given that outputs from conservation planning tools are rarely applied in the context of prioritisation across existing protected area portfolios, it is important to assess the performance of the different metrics that can be, or are, used in such circumstances. In this paper, we evaluate four simple indices, including a novel Conservation Value Index (*CVI*), that could be used to identify priority sites for investment from a network of 22 designated and candidate protected areas in the contiguous dry regions of Madagascar. We benchmark our findings against the site ranking produced using the widely-used systematic planning software Zonation [39], and consider the relative strengths and weaknesses of each approach, given the constraints of data availability that may hinder conservation decision-making.

## Materials and Methods

### Study region and taxa

Madagascar is one of the world’s top conservation priorities [40, 41]. Since 2003, it has been implementing its ‘Durban Vision’, an ambitious programme to extend the coverage of its protected area system from 1.7 million ha to 6 million ha [42, 43]. However, progress towards this goal has been hampered by the 2009–2014 political crisis, which left the country without a recognised legitimate government, leading to increased illegal wood trafficking and reduced funding for conservation [44, 45]. Prior to the Durban Vision, all Malagasy protected areas were managed by the parastatal Madagascar National Parks (MNP) and were designated as IUCN category I, II or IV, with the primary objective of conserving biodiversity [46]. In comparison, the new generation of sites is composed mainly of category III, V and VI multiple-use protected areas, managed for multiple socio-economic objectives as well as the maintenance of biodiversity, and administered by a range of actors including local community associations, non-governmental organisations (NGOs) and decentralised state authorities [47–49]. Together with the existing MNP protected areas, they form the Madagascar Protected Area System (SAPM).

The location of new protected areas has been partially informed by gap analyses and systematic conservation planning [50, 51], based purely on biodiversity data without the inclusion of cost information [43], with the aim of maximising the representation of endemic biodiversity within SAPM. Over 500 priority sites were identified, of which 93 had been granted permanent or temporary protected status by 2012 [52]. The organisation(s) responsible for each protected area, which are primarily Malagasy and international NGOs, are expected to independently source the necessary funds to ensure its long-term management. However, the Madagascar Foundation for Protected Areas and Biodiversity has been created to meet emergency shortfalls for the ‘best’ protected areas, which are characterised as such based on factors including a site’s contribution to biodiversity representation within SAPM (M. Nicoll, *pers*. *comm*.). Thus, many protected areas are essentially competing for some of their funds from the same, limited, pool of financial support. In their management plans, individual protected areas promote their importance on the basis of species richness, as well as lists of threatened and locally endemic species.

The freely available and comprehensive reptile inventory data from a range of sites in the contiguous dry regions of Madagascar make this taxonomic group a convenient case study for assessing the performance of prioritisation tools. The country’s reptile fauna is diverse, comprising almost 400 species, with 92% of these being endemic [53–55]. Many of the species are highly range restricted or micro-endemic [56, 57], and the majority are forest-dependent [55, 57, 58]: given historical deforestation trends [59], such species may depend on the effective maintenance of protected areas for their long-term survival. The dry regions of Madagascar are composed of two ‘Global 200’ priority ecoregions: the Madagascar dry forests in the west, and the Madagascar spiny desert of the south and southwest [60]. Although northern Madagascar also harbours dry forests, they are isolated from the western areas by a large band of humid forest and are excluded from this analysis due to a lack of inventory data.

### Biodiversity survey data

**Fig 1:**
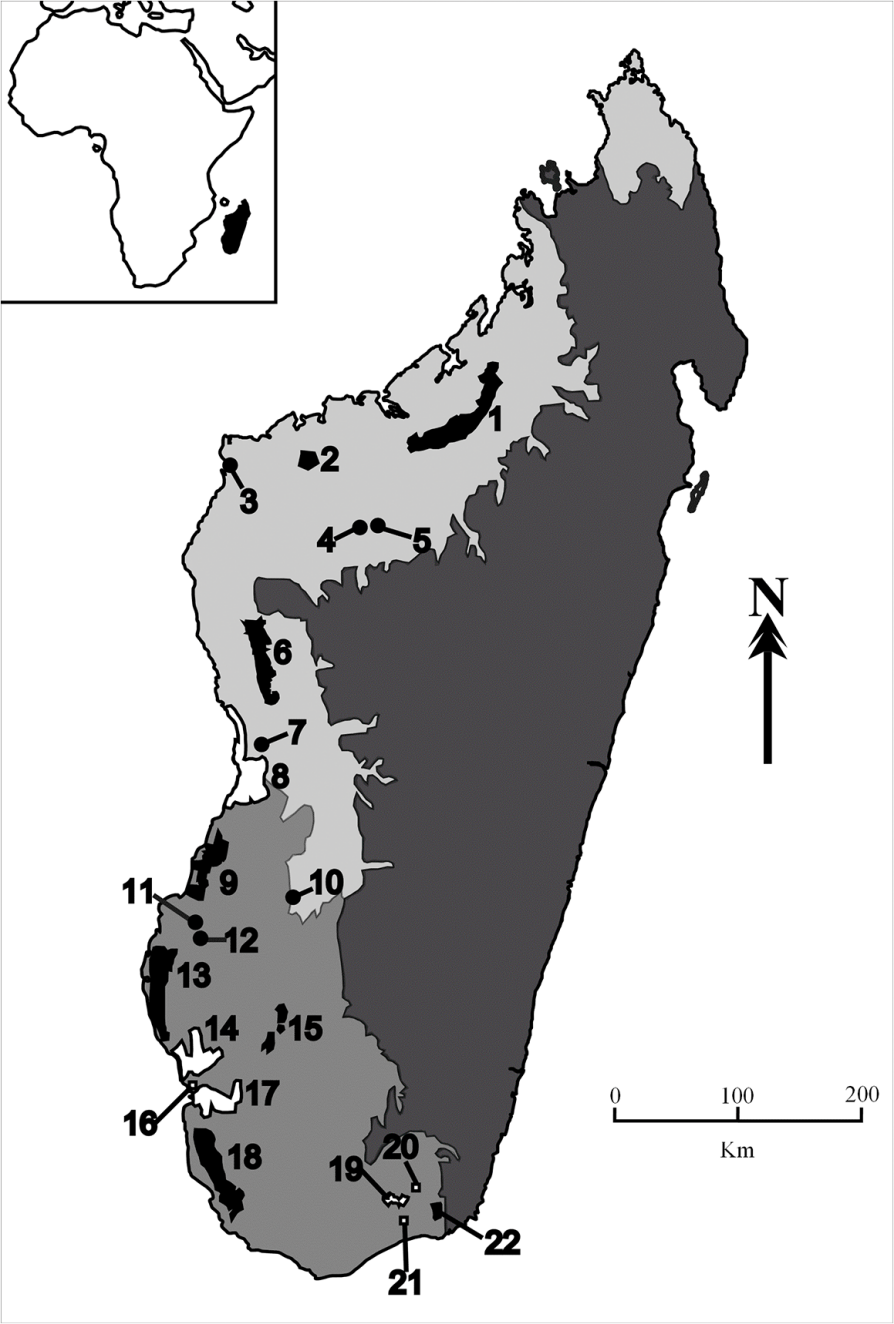
Map of Madagascar showing location of existing and candidate protected areas used in prioritisation. National parks are indicated by black polygons, new protected areas by white polygons/squares, and candidate protected areas by black circles (additional protected areas not used in analysis are not shown): 1, Ankarafantsika; 2, Namoroka; 3, Andranomanintsy; 4, Kelifely; 5, Ankara; 6, Tsingy de Bemaraha; 7, Masoarivo; 8, Menabe Antimena; 9, Kirindy Mite; 10, Makay; 11, Berento; 12, Nosy-Ambositra; 13, Mikea; 14, Ranobe PK32; 15, Zombitse-Vohibasia; 16, Tsinjoriake; 17, Amoron’i Onilahy; 18, Tsimanampetsotsa; 19, Nord Ifotaka; 20, Anadabolava-Betsimalaho; 21, Behara-Tranomaro, and; 22, Andohahela Parcel 2. The dry bioclimatic region is shown in light grey, the sub-arid region in medium grey, and the sub-humid and humid regions in dark grey (following [109]). The inset shows the position of Madagascar relative to mainland Africa.

We compiled a database of reptile inventories for both established and candidate protected areas in western and southern Madagascar (Fig 1; S1 Table), and supplemented these data with our own survey records (C. Raxworthy, *unpublished data*). All inventories used standard protocols including pit-fall traps and refuge searches (see references in S1 Table). The database included inventories of 22 sites, comprising eight national parks, seven new protected areas and seven candidate protected areas which have been identified as priority sites for future designation (Fig 1). Species taxonomy follows Glaw and Vences [58] and subsequent revisions wherever the specific identity of split taxa is unambiguous [61–64]. The database was cleaned by removing all records of species no longer considered valid (i.e. subsequently synonymised; n = 2), records that have not been described/identified to species level (n = 10), probable misidentifications (n = 5) and introduced species (n = 1).

### Simple site prioritisation indices

Sites were ranked on the basis of four simple prioritisation protocols: 1) species richness, (the number of species recorded at a site, a measure often used by decision-makers); 2) an index derived from species Red List status [65]; 3) an irreplaceability index (a key metric in systematic conservation planning); and, 4) a Conservation Value Index (*CVI*) in which species were scored on the basis of four attributes reflecting rarity and threat. For protocols 2, 3 and 4, scores were assigned to individual species and the site score (SS) was then calculated as the cumulative score of all species occurring there.

#### Protocol 1 –*Richness* (SR)

$${SS}_{SR}=r\;\left(\text{where}\;r\;\text{is the species richness of a site}\right)$$ (1)

#### Protocol 2 –*Red List Index* (RL)

Scores were assigned to species on the basis of their Red List rankings from the 2011 Global Reptile Assessment for Madagascar [65], as follows: 5, Critically Endangered (CR); 4, Endangered (EN); 3, Vulnerable (VU); 2, Near Threatened (NT); 1, Least Concern (LC). Species classified as Data Deficient (DD) (n = 9) or Not Evaluated (NE) (n = 4) were excluded on the basis that insufficient data exist to permit evaluation.

$${SS}_{RL}=\sum {RL}_{species}$$ (2)

#### Protocol 3 –*Irreplaceability* (IR)

Using a method based on Brugière and Scholte [66], where each species is weighted by the inverse of the number of protected areas in which it was recorded.

IRspecies=1n(wherenis the number of protected areas within the sample at which a species occurs) (3)

$${SS}_{IR}=\sum {IR}_{species}$$ (4)

#### Protocol 4 –*Conservation Value Index* (CVI)

Scores were assigned to each species based on four attributes that reflect relative rarity and threat, and thus extinction risk. Rarity was assessed using degree of endemism (*E*) and representation in protected areas within the study sample (*R*); for both these attributes, ‘rarer’ species score higher than widespread and well-represented species. Threat was based on hunting and collection pressure (*C*) and degradation tolerance (*T*), because these factors have a significant influence on the long-term viability of Madagascar’s reptiles [55, 57]. The relative tolerance of species to habitat modification is particularly critical [55, 67–69] as most species in Madagascar are forest dependent [57], and forest loss and degradation outside of protected areas shows no sign of reducing [59, 70]. Degradation-tolerant species may maintain viable populations in transformed environments outside protected areas [71–73] and so assumed lower conservation priority within the CVI. Hunting for domestic consumption and collection for the global pet trade affect comparatively few species, but represent the primary extinction threat to those that are targeted [55, 74, 75].

We assigned scores to each species on a five-point scale for each attribute (Table 1). For *E*, we visually estimated range thresholds using distribution maps in Glaw and Vences [58]. Watershed-based biogeographical models [76] were not used to identify micro-endemic species because they are not a good proxy for local endemism in reptiles [77]. Instead we used 10,000 km^2^ (approximately 2% of Madagascar’s land surface) as the threshold range size to distinguish between micro-endemics and endemic species restricted to a single bioclimatic region. *R* was scored on the basis of occurrences in protected areas within this study. *C* threat values were determined using CITES (Convention on International Trade in Endangered Species) listings and the literature on reptile declines in Madagascar. Scores for *T* were assigned according to a review of the literature; species for which no degradation tolerance data were available (n = 24) were assumed to be degradation intolerant (i.e. given a score of 5) on the basis of the precautionary principle.

**Table 1:** Attributes and scoring criteria used in Conservation Value Index (*CVI*) and Zonation assessments (*E*, *C* and *T* only). PA = protected area.

	Rarity factors		Threat factors	
Score	Degree of endemism (*E*)	Representation in sample PAs (*R*)	Hunting and collection pressure (*C*)	Degradation tolerance (*T*)
1	Indigenous, non-endemic species	Recorded in 12–15 PAs (n > 75%)	No known threat	Tolerant of modified or artificial habitats
2	Widespread endemic, occurring in dry and humid regions	Recorded in 8–11 PAs (45 > n < 75%)	N/A	N/A
3	Endemic to dry regions	Recorded in 4–7 PAs (20 > n < 45%)	Known threat (CITES Appendix I and II), but not likely to cause local extirpations	Tolerant of edge effects, medium-intensity degradation or secondary growth.
4	Endemic to one bioclimatic region^a^	Recorded in 2–3 PAs (10 > n < 20%)	N/A	N/A
5	Local endemic, range size estimated as < 10,000 km^2^	Recorded in only 1 PA (n < 10%)	Threat known to have caused local extirpations or severe population declines	Intolerant of low-intensity degradation

^a^ Following Cornet [109]

Since rarity and threat are likely to interact in influencing species viability, summed rarity and threat values were multiplied to produce a *CVI* score (range: 4–100).

$${CVI}_{species}=\left(E+R\right)\times \left(C+T\right)$$ (5)

$${SS}_{CVI}=\sum {CVI}_{species}$$ (6)

To test the sensitivity of *CVI* to variation in the weighting of individual attribute scores for species, we performed sensitivity analyses in which the relative weighting of each attribute was doubled.

### Zonation

In order to produce a definitive benchmark against which to compare the site prioritisation protocols, we ran an assessment using the systematic conservation planning software Zonation v3.1 [78]. Zonation is a spatial conservation prioritisation framework which is based on conservation feature distributions defined using grid cells [39]. The underlying meta-algorithm starts from the full landscape and proceeds by iterative removal of cells (sites), at each step eliminating those which result in the smallest marginal loss in conservation value. The most important cells in the landscape are thus retained until last. Subsequently, Zonation produces a hierarchical ranking of conservation priority for each cell over the entire landscape [78]. We therefore: i) converted the presence-absence data for each species into a raster grid format to identify the distribution of each species across the landscape, with each site represented by a single cell; and, ii) used the Zonation additive-benefit function removal rule which bases selection on a cell’s weighted summed occurrence value over all species [39]. With this cell removal rule, species occurrences are considered additive, so the cell with the lowest value summed across all species will be removed at each step [78]. The result is that species rich cells tend to have a higher value than cells containing fewer species.

In order to produce the most informed ranking possible, species were weighted on the basis of endemism (*E*), hunting and collection pressure (*C*) and degradation tolerance (*T*) scores from *CVI*, according to the formula: weighting = (*E*) x (*C* + *T*). Representation scores were not included in the weighting as these data are incorporated in the Zonation algorithm. Since these species weightings may bias the results to be more closely correlated with those produced by *CVI*, we also ran the Zonation analysis with all species weighted equally.

### Comparison and assessment of site prioritisation indices

We evaluated the performance of each of the four prioritisation protocols by comparing the resultant site rankings with those produced by Zonation, using Spearman’s rank correlations.

## Results

The final dataset contained 134 species distributed across 12 families: Boidae (3), Chamaeleonidae (14), Crocodylidae (1), Gekkonidae (36), Gerrhosauridae (8), Iguanidae (6), Lamprophiidae (34), Pelomedusidae (2), Podocnemididae (1), Scincidae (24), Testudinidae (3), and Typhlopidae (2).

### Species rankings

The three non-*Richness* site prioritisation protocols produced species rankings that are broadly similar, but important differences emerged for certain species (Table 2). For example, two tortoises (*Astrochelys radiata* and *Pyxis arachnoides*) ranked in the top 10% of species using *CVI* and *Red List Index*, but featured in the lower 35% of species using *Irreplaceability*. Using *CVI*, the 15 highest ranked species include four members of the order Testudines (tortoises and turtles), seven species in the family Chamaeleonidae (chameleons) and four species in the family Gekkonidae (geckos) (S2 Table).

**Table 2:** Rank of the highest and lowest scoring 20 reptile species (n = 134) from the dry regions of Madagascar according to the conservation value index (*CVI*), and compared with equivalent scores and ranks generated by the red list (*RL*) and irreplaceability (*IR*) protocols. ‘ = ‘ indicates species of equal rank.

**Highest scoring 20 species according to *CVI***	***CVI* score**	***CVI* rank**	***RL* score**	***RL* rank**	***IR* score**	***IR* rank**
*Brookesia bonsi*	80	= 1	5	= 1	1	= 1
*Brookesia decaryi*	80	= 1	4	= 7	1	= 1
*Brookesia exarmata*	80	= 1	4	= 7	1	= 1
*Brookesia perarmata*	80	= 1	4	= 7	1	= 1
*Furcifer belalandaensis*	80	= 1	5	= 1	1	= 1
*Pyxis planicauda*	80	= 1	5	= 1	1	= 1
*Erymnochelys madagascariensis*	80	= 1	5	= 1	0.5	= 37
*Furcifer nicosiai*	72	= 8	4	= 7	1	= 1
*Phelsuma breviceps*	72	= 8	3	= 19	0.33	= 59
*Uroplatus henkeli*	72	= 8	3	= 19	1	= 1
*Furcifer rhinoceratus*	72	= 8	3	= 19	1	= 1
*Astrochelys radiata*	70	= 12	5	= 1	0.17	= 92
*Pyxis arachnoides*	70	= 12	5	= 1	0.17	= 92
*Phelsuma borai*	64	= 14	0	= 122	0.5	= 37
*Uroplatus guentheri*	64	= 14	4	= 7	0.33	= 59
*Ebenavia maintimainty*	60	= 16	4	= 7	1	= 1
*Lygodactylus klemmeri*	60	= 16	2	= 37	1	= 1
*Paragehyra petiti*	60	= 16	3	= 19	1	= 1
*Pygomeles petteri*	60	= 16	4	= 7	1	= 1
*Sirenoscincus yamagishii*	60	= 16	4	= 7	1	= 1
**Lowest scoring 20 species according to *CVI***	***CVI* score**	***CVI* rank**	***RL* score**	***RL* rank**	***IR* score**	***IR* rank**
*Zonosaurus laticaudatus*	16	= 110	1	= 45	0.08	= 123
*Oplurus cyclurus*	16	= 110	1	= 45	0.09	= 117
*Oplurus cuvieri*	16	= 110	1	= 45	0.2	= 85
*Langaha madagascariensis*	16	= 110	1	= 45	0.13	= 104
*Leioheterodon madagascariensis*	16	= 110	1	= 45	0.13	= 104
*Lygodactylus tuberosus*	14	120	1	= 45	0.25	= 75
*Lygodactylus tolampyae*	12	= 121	1	= 45	0.08	= 123
*Madagascarophis colubrinus*	12	= 121	1	= 45	0.08	= 123
*Dromicodryas quadrilineatus*	12	= 121	1	= 45	0.5	= 37
*Thamnosophis lateralis*	12	= 121	1	= 45	0.33	= 59
*Furcifer verrucosus*	10	= 125	0	= 122	0.09	= 117
*Paroedura picta*	10	= 125	1	= 45	0.09	= 117
*Furcifer lateralis*	8	= 127	1	= 45	0.1	= 114
*Furcifer oustaleti*	8	= 127	1	= 45	0.09	= 117
*Chalarodon madagascariensis*	8	= 127	1	= 45	0.09	= 117
*Trachylepis elegans*	6	= 130	1	= 45	0.07	= 131
*Trachylepis gravenhorstii*	6	= 130	1	= 45	0.08	= 123
*Dromicodryas bernieri*	6	= 130	1	= 45	0.07	= 131
*Mimophis mahfalensis*	6	= 130	1	= 45	0.07	= 131
*Hemidactylus mercatorius*	4	134	1	= 45	0.08	= 128

### Site rankings

**Fig 2:**
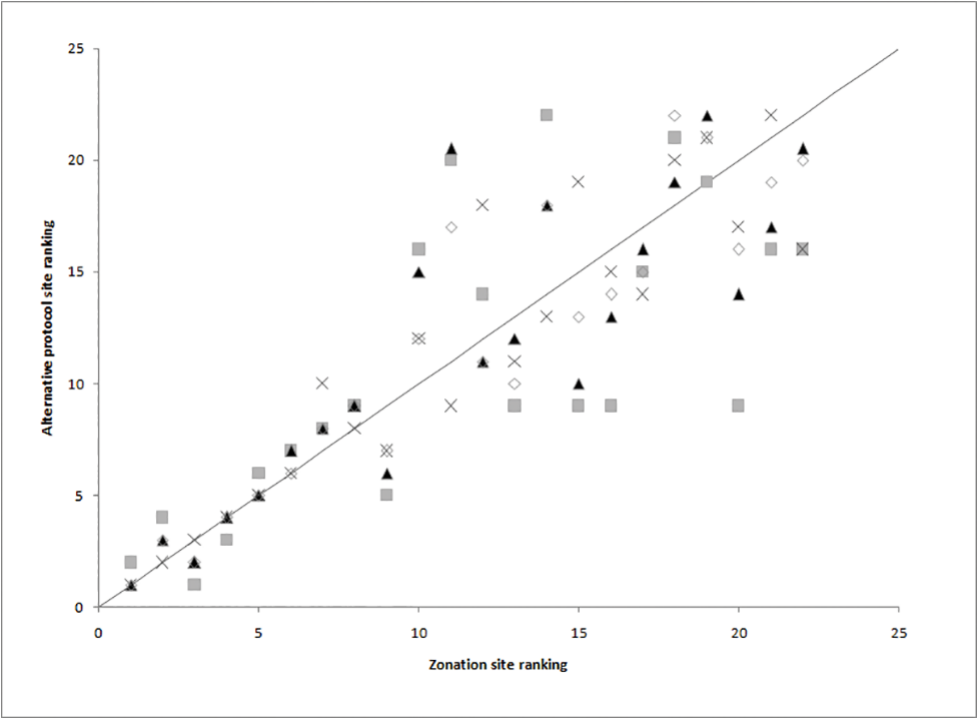
Correlation of site rankings produced by Zonation and four simple protocols: Grey squares, Richness; black triangles, Red List Index; crosses, Irreplaceability; white diamonds, Conservation Value Index (*CVI*). Solid line represents x = y.

Site species richness ranged from 17 in Kelifely to 72 in Ranobe PK32 (mean = 35.3, S.E. = 3.3, median = 30). The site rankings produced by the four prioritisation protocols are all strongly positively correlated with the output of Zonation (Table 3, Fig 2). In ascending order, the weakest correlation was between Zonation and *Richness* (*r*_*s*_ = 0.711, *p* < 0.01), followed by Zonation and *Red List Index* (*r*_*s*_ = 0.861, *p* < 0.01), Zonation and *Irreplaceability* (*r*_*s*_ = 0.920, *p* < 0.01), and Zonation and *CVI* (*r*_*s*_ = 0.927, *p* < 0.01). Inter-protocol ranking variability was greater for lower, rather than higher, ranked sites (the highest ranked site being number 1; Fig 2). The high degree of association between *CVI* and Zonation was not just an artefact of using a similar species weighting system in both protocols, as *CVI* was most strongly correlated with the outputs of Zonation when species weightings were not used (*r*_*s*_ = 0.977, *p* < 0.01). Sensitivity analyses indicated that *CVI* is relatively robust to changes in individual species attribute weightings, with correlation coefficients ranging from 0.916 to 0.932 when each of the four attribute scores were doubled (S3 Table).

**Table 3:** Site status, area, scores and ranks for 22 sites in the dry regions of Madagascar, prioritised using Zonation (*Z*) and four alternative prioritisation protocols on the basis of their reptile faunas: *SR*, species richness; *RL*, red list index; *IR*, irreplaceability; *CVI*, conservation value index; NPA, new protected area. ‘ = ‘ indicates sites of equal rank. Rankings were produced to enable comparisons to be made between methods and should not be considered as definitive prioritisations because the analyses were not designed for this purpose.

Site name	Site status	Area (km^2^)	*Z* rank	*SR* rank	*RL* Rank	*IR* Rank	*CVI* rank	*SR* score	*RL* score	*IR* score	*CVI s*core
Tsingy de Bemaraha	National Park	1567.6	1	2	1	1	1	62	96	26.8	2054
Ankarafantsika	National Park	1366.1	2	4	3	2	3	53	83	20.0	1720
Ranobe PK32	NPA	1485.5	3	1	2	3	2	72	91	15.7	1966
Mikea	National Park	1846.4	4	3	4	4	4	57	73	11.8	1544
Tsimanampesotse	National Park	2037.4	5	6	5	5	5	51	63	10.2	1342
Menabe Antimena	NPA	2111.5	6	7	7	6	6	42	55	8.7	1130
Tsinjoriake	NPA	58.6	7	8	8	10	8	34	46	6.5	884
Namoroka	National Park	224.3	8	= 9	9	8	9	30	38	7.0	708
Amoron’i Onilahy	NPA	1021.8	9	5	6	7	7	52	59	7.8	1124
Nosy-Ambositra	Candidate	-	10	= 16	15	12	12	24	31	5.2	586
Ankara	Candidate	-	11	20	= 20	9	17	19	23	6.7	508
Anadabolava-Betsimalaho	NPA	181.1	12	14	11	18	11	29	36	3.5	622
Andranomanintsy	Candidate	-	13	= 9	12	11	10	30	34	6.2	688
Kelifely	Candidate	-	14	22	18	13	18	17	25	5.1	460
Andohahela P2	National Park	129.2	15	= 9	10	19	13	30	37	3.5	572
Zombitse-Vohibasia	National Park	369.0	16	= 9	13	15	14	30	33	3.8	572
Masoarivo	Candidate	-	17	15	16	14	15	26	30	4.5	556
Berento	Candidate	-	18	21	19	20	22	18	24	3.1	386
Behara-Tranomaro	NPA	965.9	19	19	22	21	21	23	22	2.6	422
Kirindy Mite	National Park	1390.2	20	= 9	14	17	16	30	32	3.5	528
Nord Ifotaka	NPA	222.6	21	= 16	17	22	19	24	28	2.5	448
Makay	Candidate	-	22	= 16	= 20	16	20	24	23	3.6	438

Using *CVI* to compare sites of different protected status suggests that national parks (mean *CVI* = 1130.0, S.E. = 214.7, n = 8) are of greater conservation value for the reptile fauna than new protected areas (mean *CVI* = 942.3, S.E. = 203.4, n = 7) or candidate sites for future designation (mean *CVI* = 517.4, S.E. = 38.5, n = 7). This finding was consistent across all the prioritisation protocols.

## Discussion

We have carried out the first evaluation of a range of well-known and new heuristic metrics which can be used to prioritise investment across a portfolio of sites. The site rankings produced by all prioritisation protocols were strongly correlated with the outputs of Zonation due to the fact that they are partially driven by species richness; since each individual species score is positive, sites scores will increase with greater numbers of recorded species. With the exception of *Species Richness*, all protocols were consistent in the selection of the top five ranking protected areas but showed greater variability in selecting between lower-ranking sites. This suggests that, while the most important of these sites would always be targeted to receive funding, the more marginal sites may be more difficult to reliably identify, thus making the selection of an appropriate prioritisation protocol of even greater importance. Despite their similarities, variation in the performance of the different protocols, when compared to the Zonation benchmark, provides insight into the suitability of each for use in the prioritisation of protected areas for investment.

Conservation assessments are intended to inform decisions, rather than provide definitive prescriptions [33, 79]. Ideally, with any prioritisation exercise, decision-makers should use a systematic approach such as Zonation whenever they have the capacity to do so or, if this is lacking, seek to develop or ‘borrow’ the necessary expertise by collaborating with research institutions [80, 81]. In addition to providing more robust assessments, Zonation can also be used for complex modelling that is beyond the scope of heuristic metrics (e.g. incorporating future climate change scenarios into planning) [82]. Where available, data reflecting species value (such as the attributes used in the *CVI*, population viability, or other characteristics discussed below) should be integrated into assessments in order to ensure that the evidence base is as rich and robust as possible, hence we included additional information on species threat status into our *CVI* protocol and benchmark assessment. It would have been best practice to have included non-biodiversity data in the assessment explicitly, for example cost information [83–85], probability of habitat loss, and the relative effectiveness [86] of the different protected area models employed in SAPM. However, such data are unavailable for Madagascar and, therefore, they were not used in this study or in the prioritisation exercise that informed the location of new protected areas within the Durban Vision expansion [43].

However, in situations where systematic conservation assessment software cannot be used, indices can provide a transparent, repeatable evidence base to inform prioritisation decision-making, thus representing an improvement on non-systematic approaches. The simplest such index is *Species Richness*, but this metric performed relatively poorly in our analysis, and would have identified Ranobe PK32 as the most important site for reptile conservation in our sample. All other protocols consistently rank Tsingy de Bemaraha as the most valuable site, despite it harbouring only 86% as many species as Ranobe PK32, because 28% of its reptiles are locally endemic [87]. Furthermore, *Species Richness* is not an accurate indicator of conservation value [88, 89] because all species are not equal. While value can be assigned to species according to a range of criteria (e.g. genotypic [90] or phenotypic [91] distinctiveness, public preferences [92], or ecological function [93]), we differentiated between species using parameters that reflect extinction risk as this is the most urgent issue facing conservationists [94]. Understanding the threats faced by species is critical to estimating their vulnerability and thus dependence on conservation interventions [74], yet systematic conservation planning assessments do not always incorporate such data.

The richness of threatened species is often used to inform prioritisations (e.g. [37, 38]), and the strong (0.86) positive correlation between the *Red List Index* and Zonation suggests this metric may be a useful proxy measure if the necessary data are available. However, the *Red List Index* failed to account for Data Deficient and Not Evaluated species, and thus almost 10% of species in our sample were excluded from the analysis. Additionally, since the use of such an index is dependent on the availability of full, up-to-date Red List assessments, its utility will be limited for many taxonomic groups and geographical regions, given that only 2.75% of described species had been evaluated by 2010 [33].

The concept of irreplaceability is a key metric in systematic conservation assessments, and the *Irreplaceability* index performed well in comparison to Zonation. However, measures of irreplaceability alone may not adequately reflect conservation value, because some species may be widespread and occur in a number of protected areas, yet remain highly threatened. For example, the tortoises *Astrochelys radiata* and *Pyxis arachnoides* were ranked low in terms of irreplaceability as both species are present in six protected areas, but they have suffered rapid, range-wide declines in population density [95, 96] that have resulted in them being classified as Critically Endangered [97]. In addition, care is needed when dealing with species that are commonly found outside the sites being considered. For example, the gecko *Phelsuma modesta* was recorded in only one protected area and therefore ranked high in terms of irreplaceability, although its abundance in heavily modified, non-forest habitats (e.g. urban areas [58, 98]) demonstrate that it is not dependent on the effective management of protected areas for its survival. Similar problems may arise if species occur within the study region, but only at the periphery of their range (e.g. if reptiles widespread in humid eastern Madagascar occurred at sites on the edge of the dry forest). However, in the case of the current case study, this issue is mitigated by the extremely high rates of species turnover between the humid and dry regions of Madagascar [55, 58, 99]. The problem of species that appear rare in a dataset but, in reality, are not, will afflict any richness or complementarity-based analysis. In such cases, one might consider excluding these species or, alternatively, using an explicitly target-based approach to measuring irreplaceability which sets lower targets for species deemed of minimal conservation importance by planners. Nonetheless, this risks introducing an element of subjectivity into the prioritisation exercise unless these species can be systematically identified by, for instance, using *CVI*.

The strongest correlation between site rankings was produced for Zonation and *CVI*, suggesting that the latter index could be used to inform protected area prioritisation in situations where more sophisticated analyses are not feasible. The index incorporated measures of rarity (a proxy for irreplaceability) and threat (a proxy for Red List status). As it only used inventory data, which were compiled into a database of species presence, and published literature to assign attribute scores using a simple scoring system, it can be adopted by decision-makers without the need for specific technological training. Nevertheless, the data requirements for the use of *CVI* are more burdensome than for Zonation and the other indices tested, which may limit its utility. Although *CVI* performed well in prioritising the forests of Madagascar’s dry regions for reptile conservation, additional case studies are needed to further examine its functionality. In particular, the approach may be most appropriate for dealing with a small number of pre-identified sites (e.g. prioritising across an existing protected area portfolio), rather than for carrying out a conservation assessments which might seek to prioritise among many (i.e. hundreds or even thousands) of localities to optimise the establishment of new protected areas.

Our study was designed to investigate the performance of different protocols in prioritising amongst a portfolio of sites, and we do not intend it to serve as a definitive prioritisation of protected areas within the study region. Biases may have arisen due to the variation in survey effort undertaken at each site, which ranges over two orders of magnitude in terms of survey duration (S1 Table). However, most conservation decision-making is not only urgent but characterised by an imperfect evidence base. This is especially true given the expense of biodiversity surveys in tropical developing countries [100] and the rapidly diminishing returns from increased inventorying [101]. As such it is more prudent to make decisions based on available information and invest in action, as opposed to further data collection [102–104]. In addition, spatial patterns of reptile distributions and associated conservation value may not reflect those of other species groups, thus limiting the utility of a single-group assessment. While patterns of cross-taxon distributions are highly congruent at a global scale [105], this is not always the case at smaller scales within Madagascar [50, 77]. Thus the inclusion of data on multiple taxonomic groups would be required to provide a more robust prioritisation of investment across existing and candidate protected areas in the study region.

Despite these caveats, our results provide new insights into the relative conservation importance of candidate and existing protected areas in western and southern Madagascar. As a group, the established generation of national parks are more valuable for reptile conservation than both the Durban Vision generation of new protected areas and hitherto unprotected candidate sites; national parks comprise four of the five highest ranking sites for all protocols apart from *Species Richness*, and only one national park is in the bottom-ranked 30%. Two candidate sites, Nosy-Ambositra and Ankara, rank amongst the top 50% of sites using Zonation, and therefore warrant consideration for future protected area establishment. Currently, many of Madagascar’s national parks currently lack sufficient resources [52] to prevent deforestation within their boundaries [20, 70, 106]. Given the fact that management effectiveness can be improved with even modest increases in funding [17], the analyses suggest that available finances may be best invested in the existing national park system and the Durban Vision generation of new protected areas, rather than designating and managing additional new protected areas. A robust test of this question would need to factor in the relative costs and marginal conservation gains of funding the establishment of new protected areas versus the management of established sites [85], and should be considered a priority question for funders of national protected area systems worldwide.

## Conclusions

The prioritisation of protected areas for investment is important if we are to maximise the effectiveness of protected area networks for biodiversity conservation. However, many conservation assessments and systematic conservation planning exercises do not contribute to the implementation of conservation action [32, 107, 108]. Given that management is limited by insufficient financial resources, it is all the more critical that available funds are targeted towards the most important sites. While sophisticated analytical tools can and should be used to inform such investment decisions, decision-makers often lack the capacity to use them, or choose not to for other reasons. Instead, they frequently rely on non-transparent, subjective processes or simple measures such as species richness or the number of threatened species. It is therefore important to understand how such metrics can perform and in what circumstances they should be used. Our analysis suggests that some heuristic indices can provide a transparent framework to support evidence-based decision-making by practitioners, although their performance is variable and partially dependent on the amount of information required to use them. Our *CVI*, which incorporates measures of rarity and threat for individual species, appears to provide a useful alternative to more sophisticated systematic conservation planning tools, and emphasises the benefits of integrating species-specific data into conservation assessments.

## Supporting Information

S1 Table Sources of reptile data for the 22 sites in the dry regions of Madagascar used as a case study system to compare four different site prioritisation protocols and Zonation (NPA = new protected area established since 2003). (DOC)

S2 Table Attribute scores assigned to 134 reptile species found across 22 sites in the dry regions of Madagascar, used to calculate the conservation value index (*CVI*). (DOC)

S3 Table The site prioritisation rankings according to the conservation value index (*CVI*) protocol, following sensitivity analyses where each of individual species attribute scores were doubled. (DOC)
