# Supplementary material for: Comparing Methods for Prioritising Protected Areas for Investment: A Case Study Using Madagascar’s Dry Forest Reptiles
Source: PLoS One. 2015 Jul 10;10(7):e0132803. doi: 10.1371/journal.pone.0132803 (PMC4498610; doi:10.1371/journal.pone.0132803)
Supplement: S1 Table — (DOC) [file pone.0132803.s001.doc]

**Supporting Information**

**S1 Table.** Sources of reptile data for the 22 sites in the dry regions of Madagascar used as a case study system to compare four different site prioritisation protocols and Zonation (NPA = new protected area established since 2003).

| **Site number** | **Site name** | **Protected area status** | **Data sources** | **Number of localities**  **surveyed** | **Total survey duration (days)** |
| --- | --- | --- | --- | --- | --- |
| 1 | Ankarafantsika | National Park | [1,2] | 5 | 38 |
| 2 | Namoroka | National Park | [1] | 2 | 11 |
| 3 | Andranomanintsy | Candidate | [1] | 1 | 6 |
| 4 | Kelifely | Candidate | [3] | 1 | 7 |
| 5 | Ankara | Candidate | [3] | 1 | 7 |
| 6 | Tsingy de Bemaraha | National Park | [1,4,5] | 12 | 136 |
| 7 | Masoarivo | Candidate | [1] | 2 | 13 |
| 8 | Menabe Antimena | NPA | [1,6^a^] | 2 | 14 |
| 9 | Kirindy Mite | National Park | [1] | 4 | 28 |
| 10 | Makay | Candidate | [3] | 2 | 16 |
| 11 | Berento | Candidate | [3] | 1 | 7 |
| 12 | Nosy-Ambositra | Candidate | [3] | 1 | 8 |
| 13 | Mikea | National Park | [1,7] | 6 | 40 |
| 14 | Ranobe PK32 | NPA | [8,9] | 3 | 578 |
| 15 | Zombitse-Vohibasia | National Park | [10,11] | 5 | 36 |
| 16 | Tsinjoriake | NPA | [12] | 2 | 16 |
| 17 | Amoron’i Onilahy | NPA | [13] | 5 | 378 |
| 18 | Tsimanampetsotsa | National Park | [1,14] | 3 | 22 |
| 19 | Nord Ifotaka | NPA | [1] | 1 | 6 |
| 20 | Anadabolava-Betsimalaho | NPA | [1] | 1 | 7 |
| 21 | Behara-Tranomaro | NPA | [1] | 1 | 7 |
| 22 | Andohahela Parcel 2 | National Park | [15] | 1 | 8 |

^a^ Survey duration not known and therefore not accounted for in relevant column

**References**

1. Raselimanana AP (2008) Herpétofaune des forêts sèches malgaches. In: Goodman SM, Wilmé L, editors. Les forêts sèches de Madagascar. Malagasy Nature 1: 46-75.
2. Ramanamanjato JB, Rabibisoa N (2002). Evaluation rapide de la diversité biologique des reptiles et amphibiens de la Réserve Naturelle Intégrale d’Ankarafantsika. In: Alonso LE, Schulenberg TS, Radilofe S, Missa O, editors.  Une évaluation biologique de la Réserve Naturelle Intégrale d’Ankarafantsika, Madagascar. RAP Bulletin of Biological Assessment 23: 98-193
3. Rakotondravony HA, Goodman SM (2011) Rapid herpetofaunal surveys within five isolated forests on sedimentary rock in western Madagascar. Herpetol. Conserv. Biol. 6: 297-311.
4. ANGAP (Association Nationale pour la Gestion des Aires Protégées) (2003). Plan de gestion de la conservation du complexe d’Aires Protégées Tsingy de Bemaraha. Antananarivo: ANGAP. 81 p.
5. Bora P, Randrianantoandro JC, Randrianavelona R, Hantalalaina EF, Andriantsimanarilafy RR, et al. (2010) Amphibians and reptiles of the Tsingy de Bemaraha Plateau, Western Madagascar: Checklist, biogeography and conservation. Herpetol. Conserv. Biol. 5 : 111-125.
6. Bloxam QMC, Behler JL, Rakotovao ER, Randriamahazo HJAR, Hayes KT, et al. (1996) Effects of logging on the reptile fauna of the Kirindy forest with special emphasis on the Flat-tailed tortoise (*Pyxis planicauda*). In: Ganzhorn JU, Sorg JP, editors. Ecology and economy of a tropical dry forest in Madagascar. Primate Report 46: 189-201
7. Raselimanana AP (2004) l’Herpétofaune de la forêt de Mikea. In: Raselimanana AP, Goodman SM, editors. Inventaire floristique et faunistique de la forêt des Mikea: Paysage écologique et diversité biologique d’une préoccupation majeure pour la conservation. Recherches pour le Développement, Série Sciences Biologiques 21: 37-52.
8. D’Cruze NC, Sabel JA (2005) The reptiles of the Southern Mikea Forest, Madagascar. Herpetol. Bull. 93: 2-8.
9. Thomas H, Kidney D, Rubio P, Fanning E (2006) The Southern Mikea: A biodiversity survey. London: Frontier. 93 p.
10. Raxworthy CJ, Ramanamanjato JB, Raselimanana A (1994) Les reptiles et les amphibiens. In: Goodman SM, Langrand O, editors. Inventaire biologique de la forêt de Zombitse. Recherches pour le Développement, Série Sciences Biologiques No. Spécial: 41–57
11. Goodman SM, Ramanamanjato JB, Raselimanana AP (1997) Les amphibiens et les reptiles. In: Langrand O, Goodman SM, editors. Inventaire biologique forêts de Vohibasia et Isoky-Vohimena. Recherches pour le Développement, Série Sciences Biologiques 12: 110–130.
12. Raxworthy CJ (1995) Field survey of amphibians, reptiles and small mammals from the Toliara region, Madagascar, Oct. 5–30 1995. Michigan: University of Michigan. 14 p.
13. D’Cruze N, Olsonn A, Henson D, Kumar S, Emmett D (2009) The amphibians and reptiles of the lower Onilahy river valley, a temporary protected area in southern Madagascar. Herpetol. Conserv. Biol. 4: 62-79.
14. Goodman SM, Raherilalao MJ, Rakotondravony D, Raselimanana A, Razakarivony HV, et al. (2002) Inventaire des vertébrés du Parc National de Tsimanampetsotsa (Toliara). Akon’ny Ala 28: 1-36.
15. Nussbaum RA, Raxworthy CJ, Raselimanana AP, Ramanamanjato JB (1999) Amphibians and reptiles of the Réserve Naturelle Intégrale d’Andohahela, Madagascar. Fieldiana Zoology 94: 155-173.
