# Supplementary material for: Comparing Methods for Prioritising Protected Areas for Investment: A Case Study Using Madagascar’s Dry Forest Reptiles
Source: PLoS One. 2015 Jul 10;10(7):e0132803. doi: 10.1371/journal.pone.0132803 (PMC4498610; doi:10.1371/journal.pone.0132803)
Supplement: S2 Table — (DOC) [file pone.0132803.s002.doc]

**Supporting Information**

**S2 Table.** Attribute scores assigned to 134 reptile species found across 22 sites in the dry regions of Madagascar, used to calculate the conservation value index (*CVI*).

| **Family and species** | **Endemism score (*E*)** | **Representation score (*R*)** | **Rarity score**  **(*E + R*)** | **Hunting and collection score (*C*)** | **Degradation tolerance score (*T*)** | **Threat score (*C+T*)** | **Conservation value index (*CVI*) score** |
| --- | --- | --- | --- | --- | --- | --- | --- |
| **GEKKONIDAE** |  |  |  |  |  |  |  |
| *Blaesodactylus sakalava* | 3 | 1 | ***4*** | 1 | 5 | ***6*** | **24** |
| *Ebenavia maintimainty* | 5 | 5 | ***10*** | 1 | 5 | ***6*** | **60** |
| *Geckolepis maculata* | 2 | 4 | ***6*** | 1 | 3 | ***4*** | **24** |
| *Geckolepis polylepis* | 4 | 4 | ***8*** | 1 | 5 | ***6*** | **48** |
| *Geckolepis typica* | 3 | 1 | ***4*** | 1 | 5 | ***6*** | **24** |
| *Hemidactylus mercatorius* | 1 | 1 | ***2*** | 1 | 1 | ***2*** | **4** |
| *Lygodactylus heterurus* | 3 | 5 | ***8*** | 1 | 1 | ***2*** | **16** |
| *Lygodactylus klemmeri* | 5 | 5 | ***10*** | 1 | 5 | ***6*** | **60** |
| *Lygodactylus tolampyae* | 2 | 1 | ***3*** | 1 | 3 | ***4*** | **12** |
| *Lygodactylus tuberosus* | 4 | 3 | ***7*** | 1 | 1 | ***2*** | **14** |
| *Lygodactylus verticillatus* | 4 | 4 | ***8*** | 1 | 1 | ***2*** | **16** |
| *Matoatoa brevipes* | 4 | 4 | ***8*** | 1 | 5 | ***6*** | **48** |
| *Paragehyra petiti* | 5 | 5 | ***10*** | 1 | 5 | ***6*** | **60** |
| *Paroedura androyensis* | 4 | 3 | ***7*** | 1 | 5 | ***6*** | **42** |
| *Paroedura bastardi* | 3 | 1 | ***4*** | 1 | 3 | ***4*** | **16** |
| *Paroedura homalorhina* | 4 | 5 | ***9*** | 1 | 5 | ***6*** | **54** |
| *Paroedura karstophila* | 4 | 4 | ***8*** | 1 | 5 | ***6*** | **48** |
| *Paroedura maingoka* | 5 | 5 | ***10*** | 1 | 3 | ***4*** | **40** |
| *Paroedura oviceps* | 3 | 4 | ***7*** | 1 | 5 | ***6*** | **42** |
| *Paroedura picta* | 3 | 2 | ***5*** | 1 | 1 | ***2*** | **10** |
| *Paroedura stumpffi* | 3 | 4 | ***7*** | 1 | 3 | ***4*** | **28** |
| *Paroedura tanjaka* | 4 | 4 | ***8*** | 1 | 3 | ***4*** | **32** |
| *Paroedura vahiny* | 3 | 3 | ***6*** | 1 | 5 | ***6*** | **36** |
| *Paroedura vazimba* | 4 | 5 | ***9*** | 1 | 5 | ***6*** | **54** |
| *Phelsuma abbotti* | 1 | 4 | ***5*** | 3 | 1 | ***4*** | **20** |
| *Phelsuma borai* | 4 | 4 | ***8*** | 3 | 5 | ***8*** | **64** |
| *Phelsuma breviceps* | 5 | 4 | ***9*** | 3 | 5 | ***8*** | **72** |
| *Phelsuma dubia* | 1 | 5 | ***6*** | 3 | 1 | ***4*** | **24** |
| *Phelsuma kochi* | 4 | 3 | ***7*** | 3 | 3 | ***6*** | **42** |
| *Phelsuma lineata* | 2 | 5 | ***7*** | 3 | 3 | ***6*** | **42** |
| *Phelsuma modesta* | 4 | 5 | ***9*** | 3 | 1 | ***4*** | **36** |
| *Phelsuma mutabilis* | 3 | 1 | ***4*** | 3 | 1 | ***4*** | **16** |
| *Phelsuma standingi* | 4 | 3 | ***7*** | 3 | 3 | ***6*** | **42** |
| *Uroplatus ebenaui* | 3 | 4 | ***7*** | 3 | 5 | ***8*** | **56** |
| *Uroplatus guentheri* | 4 | 4 | ***8*** | 3 | 5 | ***8*** | **64** |
| *Uroplatus henkeli* | 4 | 5 | ***9*** | 3 | 5 | ***8*** | **72** |
| **SCINCIDAE** |  |  |  |  |  |  |  |
| *Amphiglossus andranovahensis* | 4 | 4 | ***8*** | 1 | 5 | ***6*** | **48** |
| *Amphiglossus ornaticeps* | 2 | 2 | ***4*** | 1 | 3 | ***4*** | **16** |
| *Amphiglossus reticulatus* | 3 | 4 | ***7*** | 1 | 3 | ***4*** | **28** |
| *Amphiglossus splendidus* | 2 | 5 | ***7*** | 1 | 5 | ***6*** | **42** |
| *Androngo trivittatus* | 4 | 4 | ***8*** | 1 | 3 | ***4*** | **32** |
| *Cryptoblepharus boutonii* | 1 | 4 | ***5*** | 1 | 3 | ***4*** | **20** |
| *Madascincus igneocaudatus* | 3 | 2 | ***5*** | 1 | 5 | ***6*** | **30** |
| *Madascincus intermedius* | 2 | 3 | ***5*** | 1 | 5 | ***6*** | **30** |
| *Pygomeles braconnieri* | 5 | 4 | ***9*** | 1 | 3 | ***4*** | **36** |
| *Pygomeles petteri* | 5 | 5 | ***10*** | 1 | 5 | ***6*** | **60** |
| *Sirenoscincus yamagishii* | 5 | 5 | ***10*** | 1 | 5 | ***6*** | **60** |
| *Trachylepis aureopunctata* | 3 | 2 | ***5*** | 1 | 3 | ***4*** | **20** |
| *Trachylepis dumasi* | 3 | 2 | ***5*** | 1 | 5 | ***6*** | **30** |
| *Trachylepis elegans* | 2 | 1 | ***3*** | 1 | 1 | ***2*** | **6** |
| *Trachylepis gravenhorstii* | 2 | 1 | ***3*** | 1 | 1 | ***2*** | **6** |
| *Trachylepis tandrefana* | 4 | 4 | ***8*** | 1 | 3 | ***4*** | **32** |
| *Trachylepis vato* | 2 | 3 | ***5*** | 1 | 3 | ***4*** | **20** |
| *Trachylepis vezo* | 5 | 4 | ***9*** | 1 | 5 | ***6*** | **54** |
| *Trachylepis volamenaloha* | 5 | 5 | ***10*** | 1 | 5 | ***6*** | **60** |
| *Voeltzkowia fierinensis* | 4 | 4 | ***8*** | 1 | 5 | ***6*** | **48** |
| *Voeltzkowia lineata* | 4 | 3 | ***7*** | 1 | 3 | ***4*** | **28** |
| *Voeltzkowia mira* | 5 | 5 | ***10*** | 1 | 5 | ***6*** | **60** |
| *Voeltzkowia petiti* | 5 | 4 | ***9*** | 1 | 5 | ***6*** | **54** |
| *Voeltzkowia rubrocaudata* | 3 | 3 | ***6*** | 1 | 5 | ***6*** | **36** |
| **GERRHOSAURIDAE** |  |  |  |  |  |  |  |
| *Tracheloptychus madagascariensis* | 4 | 2 | ***6*** | 1 | 3 | ***4*** | **24** |
| *Tracheloptychus petersi* | 5 | 4 | ***9*** | 1 | 5 | ***6*** | **54** |
| *Zonosaurus bemaraha* | 5 | 5 | ***10*** | 1 | 5 | ***6*** | **60** |
| *Zonosaurus karsteni* | 3 | 3 | ***6*** | 1 | 3 | ***4*** | **24** |
| *Zonosaurus laticaudatus* | 3 | 1 | ***4*** | 1 | 3 | ***4*** | **16** |
| *Zonosaurus maramaintso* | 5 | 5 | ***10*** | 1 | 5 | ***6*** | **60** |
| *Zonosaurus quadrilineatus* | 5 | 4 | ***9*** | 1 | 3 | ***4*** | **36** |
| *Zonosaurus trilineatus* | 4 | 4 | ***8*** | 1 | 3 | ***4*** | **32** |
| **CHAMAELEONIDAE** |  |  |  |  |  |  |  |
| *Brookesia bonsi* | 5 | 5 | ***10*** | 3 | 5 | ***8*** | **80** |
| *Brookesia brygooi* | 3 | 3 | ***6*** | 3 | 3 | ***6*** | **36** |
| *Brookesia decaryi* | 5 | 5 | ***10*** | 3 | 5 | ***8*** | **80** |
| *Brookesia exarmata* | 5 | 5 | ***10*** | 3 | 5 | ***8*** | **80** |
| *Brookesia perarmata* | 5 | 5 | ***10*** | 3 | 5 | ***8*** | **80** |
| *Furcifer angeli* | 4 | 4 | ***8*** | 3 | 3 | ***6*** | **48** |
| *Furcifer antimena* | 5 | 4 | ***9*** | 3 | 3 | ***6*** | **54** |
| *Furcifer belalandaensis* | 5 | 5 | ***10*** | 3 | 5 | ***8*** | **80** |
| *Furcifer labordi* | 3 | 3 | ***6*** | 3 | 5 | ***8*** | **48** |
| *Furcifer lateralis* | 2 | 2 | ***4*** | 1 | 1 | ***2*** | **8** |
| *Furcifer nicosiai* | 4 | 5 | ***9*** | 3 | 5 | ***8*** | **72** |
| *Furcifer oustaleti* | 2 | 2 | ***4*** | 1 | 1 | ***2*** | **8** |
| *Furcifer rhinoceratus* | 4 | 5 | ***9*** | 3 | 5 | ***8*** | **72** |
| *Furcifer verrucosus* | 3 | 2 | ***5*** | 1 | 1 | ***2*** | **10** |
| **IGUANIDAE** |  |  |  |  |  |  |  |
| *Chalarodon madagascariensis* | 2 | 2 | ***4*** | 1 | 1 | ***2*** | **8** |
| *Oplurus cuvieri* | 1 | 3 | ***4*** | 1 | 3 | ***4*** | **16** |
| *Oplurus cyclurus* | 2 | 2 | ***4*** | 1 | 3 | ***4*** | **16** |
| *Oplurus fierinensis* | 5 | 4 | ***9*** | 1 | 3 | ***4*** | **36** |
| *Oplurus quadrimaculatus* | 3 | 3 | ***6*** | 1 | 3 | ***4*** | **24** |
| *Oplurus saxicola* | 4 | 3 | ***7*** | 1 | 3 | ***4*** | **28** |
| **LAMPROPHIIDAE** |  |  |  |  |  |  |  |
| *Alluaudina bellyi* | 3 | 5 | ***8*** | 1 | 5 | ***6*** | **48** |
| *Compsophis albiventris* | 3 | 5 | ***8*** | 1 | 5 | ***6*** | **48** |
| *Dromicodryas bernieri* | 2 | 1 | ***3*** | 1 | 1 | ***2*** | **6** |
| *Dromicodryas quadrilineatus* | 2 | 4 | ***6*** | 1 | 1 | ***2*** | **12** |
| *Heteroliodon lava* | 4 | 5 | ***9*** | 1 | 5 | ***6*** | **54** |
| *Heteroliodon occipitalis* | 3 | 2 | ***5*** | 1 | 3 | ***4*** | **20** |
| *Ithycyphus miniatus* | 3 | 4 | ***7*** | 1 | 5 | ***6*** | **42** |
| *Ithycyphus oursi* | 3 | 3 | ***6*** | 1 | 5 | ***6*** | **36** |
| *Langaha alluaudi* | 3 | 4 | ***7*** | 1 | 5 | ***6*** | **42** |
| *Langaha madagascariensis* | 2 | 2 | ***4*** | 1 | 3 | ***4*** | **16** |
| *Leioheterodon geayi* | 3 | 2 | ***5*** | 1 | 3 | ***4*** | **20** |
| *Leioheterodon madagascariensis* | 2 | 2 | ***4*** | 1 | 3 | ***4*** | **16** |
| *Leioheterodon modesta* | 3 | 2 | ***5*** | 1 | 3 | ***4*** | **20** |
| *Liophidium apperti* | 4 | 3 | ***7*** | 1 | 5 | ***6*** | **42** |
| *Liophidium chabaudi* | 5 | 4 | ***9*** | 1 | 3 | ***4*** | **36** |
| *Liophidium maintikibo* | 5 | 5 | ***10*** | 1 | 5 | ***6*** | **60** |
| *Liophidium therezieni* | 3 | 5 | ***8*** | 1 | 5 | ***6*** | **48** |
| *Liophidium torquatum* | 2 | 3 | ***5*** | 1 | 3 | ***4*** | **20** |
| *Liophidium trilineatum* | 4 | 4 | ***8*** | 1 | 5 | ***6*** | **48** |
| *Liophidium vaillanti* | 3 | 3 | ***6*** | 1 | 3 | ***4*** | **24** |
| *Lycodryas citrinus* | 4 | 4 | ***8*** | 1 | 5 | ***6*** | **48** |
| *Lycodryas granuliceps* | 3 | 5 | ***8*** | 1 | 3 | ***4*** | **32** |
| *Lycodryas inornatus* | 4 | 5 | ***9*** | 1 | 5 | ***6*** | **54** |
| *Lycodryas pseudogranuliceps* | 3 | 3 | ***6*** | 1 | 5 | ***6*** | **36** |
| *Madagascarophis colubrinus* | 2 | 1 | ***3*** | 1 | 3 | ***4*** | **12** |
| *Madagascarophis meridionalis* | 3 | 3 | ***6*** | 1 | 3 | ***4*** | **24** |
| *Madagascarophis ocellatus* | 4 | 3 | ***7*** | 1 | 3 | ***4*** | **28** |
| *Mimophis mahfalensis* | 2 | 1 | ***3*** | 1 | 1 | ***2*** | **6** |
| *Phisalixella tulearensis* | 3 | 4 | ***7*** | 1 | 3 | ***4*** | **28** |
| *Phisalixella variabilis* | 5 | 4 | ***9*** | 1 | 5 | ***6*** | **54** |
| *Pseudoxyrhopus kely* | 3 | 5 | ***8*** | 1 | 5 | ***6*** | **48** |
| *Pseudoxyrhopus quinquelineatus* | 2 | 3 | ***5*** | 1 | 5 | ***6*** | **30** |
| *Thamnosophis lateralis* | 2 | 4 | ***6*** | 1 | 1 | ***2*** | **12** |
| *Thamnosophis mavotenda* | 5 | 5 | ***10*** | 1 | 5 | ***6*** | **60** |
| **BOIDAE** |  |  |  |  |  |  |  |
| *Acrantophis dumerili* | 3 | 2 | ***5*** | 3 | 1 | ***4*** | **20** |
| *Acrantophis madagascariensis* | 2 | 4 | ***6*** | 3 | 1 | ***4*** | **24** |
| *Sanzinia madagascariensis* | 2 | 3 | ***5*** | 3 | 1 | ***4*** | **20** |
| **TYPHLOPIDAE** |  |  |  |  |  |  |  |
| *Typhlops arenarius* | 3 | 2 | ***5*** | 1 | 3 | ***4*** | **20** |
| *Typhlops decorsei* | 3 | 3 | ***6*** | 1 | 3 | ***4*** | **24** |
| **PODOCNEMIDIDAE** |  |  |  |  |  |  |  |
| *Erymnochelys madagascariensis* | 4 | 4 | ***8*** | 5 | 5 | ***10*** | **80** |
| **PELOMEDISUDAE** |  |  |  |  |  |  |  |
| *Pelomedusa subrufa* | 1 | 2 | ***3*** | 3 | 3 | ***6*** | **18** |
| *Pelusios castanoides* | 1 | 3 | ***4*** | 3 | 3 | ***6*** | **24** |
| **TESTUDINIDAE** |  |  |  |  |  |  |  |
| *Astrochelys radiata* | 4 | 3 | ***7*** | 5 | 5 | ***10*** | **70** |
| *Pyxis arachnoides* | 4 | 3 | ***7*** | 5 | 5 | ***10*** | **70** |
| *Pyxis planicauda* | 5 | 5 | ***10*** | 3 | 5 | ***8*** | **80** |
| **CROCODYLIDAE** |  |  |  |  |  |  |  |
| *Crocodylus niloticus* | 1 | 3 | ***4*** | 3 | 3 | ***6*** | **24** |
