# Supplementary material for: Comparing Methods for Prioritising Protected Areas for Investment: A Case Study Using Madagascar’s Dry Forest Reptiles
Source: PLoS One. 2015 Jul 10;10(7):e0132803. doi: 10.1371/journal.pone.0132803 (PMC4498610; doi:10.1371/journal.pone.0132803)
Supplement: S3 Table — (DOC) [file pone.0132803.s003.doc]

**Supporting Information**

**S3 Table.** The site prioritisation rankings according to the conservation value index (*CVI*) protocol, following sensitivity analyses where each of individual species attribute scores were doubled.

|  | **Original *CVI*** | **Endemism (*E*) x 2** | **Representation (*R*) x 2** | **Hunting and collection (*C*)**  **x 2** | **Degradation tolerance (*T*)**  **x 2** |
| --- | --- | --- | --- | --- | --- |
| Tsingy de Bemaraha | 1 | 1 | 1 | 1 | 1 |
| Ranobe PK32 | 2 | 2 | 2 | 2 | 2 |
| Ankarafantsika | 3 | 3 | 3 | 3 | 3 |
| Mikea | 4 | 4 | 4 | 4 | 4 |
| Tsimanampetsotsa | 5 | 5 | 5 | 5 | 5 |
| Menabe Antimena | 6 | 6 | 6 | 7 | 6 |
| Amoron'i Onilahy | 7 | 7 | 7 | 6 | 7 |
| Tsinjoriake | 8 | 8 | 8 | 8 | 8 |
| Namoroka | 9 | 9 | 9 | 9 | 9 |
| Andranomanintsy | 10 | 10 | 10 | 10 | 10 |
| Anadabolava-Betsimalaho | 11 | 11 | 11 | 11 | 11 |
| Nosy-Ambositra | 12 | 13 | 12 | 12 | 13 |
| Andohahela P2 | 13 | 12 | 15 | 14 | 12 |
| Zombitse-Vohibasia | 14 | 14 | 13 | 13 | 14 |
| Masoarivo | 15 | 15 | 14 | 15 | 15 |
| Kirindy Mite | 16 | 16 | 17 | 16 | 16 |
| Ankara | 17 | 17 | 16 | 17 | 17 |
| Kelifely | 18 | 19 | 18 | 18 | 18 |
| Nord Ifotaka | 19 | 18 | 20 | 19 | 19 |
| Makay | 20 | 21 | 19 | 20 | 20 |
| Behara-Tranomaro | 21 | 20 | 21 | 21 | 21 |
| Berento | 22 | 22 | 22 | 22 | 22 |
| **Correlation with Zonation**  **(Spearman’s rank)** | **0.927** | **0.916** | **0.932** | **0.922** | **0.921** |
